# EHMTI-0084. Resting state functional connectivity abnormalities in pediatric patients with migraine

**DOI:** 10.1186/1129-2377-15-S1-B22

**Published:** 2014-09-18

**Authors:** R Messina, MA Rocca, B Colombo, E De Meo, A Falini, G Comi, M Filippi

**Affiliations:** 1 Neuroimaging Research Unit and Dept. of Neurology, Scientific Institute and University Hospital San Raffaele, Milan, Italy; 2 Dept. of Neurology, Scientific Institute and University Hospital San Raffaele, Milan, Italy; 3 Dept. of Neuroradiology, Scientific Institute and University Hospital San Raffaele, Milan, Italy

## Introduction

Previous resting state (RS) functional magnetic resonance imaging (fMRI) studies in adult patients with migraine have demonstrated abnormal functional connectivity (FC) of brain networks involved in pain processing, including the default mode (DMN), the salience (SN) and the executive control (ECN) network.

## Aims

To explore abnormalities of brain RS FC in pediatric patients with migraine and their correlation with patients’ clinical characteristics.

## Methods

Using a 3.0 Tesla scanner, RS fMRI scans were acquired from 13 pediatric migraine patients and 15 age-matched controls. Independent component analysis and a template-matching procedure were used to identify the DMN, ECN, working memory networks (WMN), SN, sensorimotor (SM), auditory and visual (VN) networks. Within-group and between-group RS FC comparisons and analysis of correlation were performed using SPM8.

## Results

Compared to controls, pediatric migraine patients had an increased RS FC of the orbito-frontal, middle and posterior cingulate gyrus of the DMN and WMNs, the inferior temporal gyrus of the ECN, the rolandic operculum and lingual gyrus of the WMNs and the postcentral gyrus of the VN. They also experienced a decreased RS FC of the anterior cingulum of the SN, the middle temporal gyrus and cerebellar vermis of the WMNs and the superior temporal gyrus of the SM. Altered RS FC of the temporal lobes of the ECN and WMN was correlated with disease duration and attack frequency.

## Conclusions

In pediatric migraine patients, distributed abnormalities of brain RS FC occur and engage not only pain-facilitating and pain-inhibiting regions, but also areas involved in executive processes.

No conflict of interest.

